# The non-dosage compensated *Lsp1*α gene of *Drosophila melanogaster *escapes acetylation by MOF in larval fat body nuclei, but is flanked by two dosage compensated genes

**DOI:** 10.1186/1471-2199-8-35

**Published:** 2007-05-19

**Authors:** Vikki M Weake, Maxwell J Scott

**Affiliations:** 1 Centre for Functional Genomics, Institute of Molecular BioSciences, Massey University, Private Bag 11 222, Palmerston North, New Zealand

## Abstract

**Background:**

In *Drosophila melanogaster *dosage compensation of most X-linked genes is mediated by the male-specific lethal (MSL) complex, which includes MOF. MOF acetylates histone H4 at lysine 16 (H4K16ac). The X-linked *Larval serum protein one *α (*Lsp1*α) gene has long been known to be not dosage compensated. Here we have examined possible explanations for why the *Lsp1*α gene is not dosage compensated.

**Results:**

Quantitative RNase protection analysis showed that the genes flanking *Lsp1*α are expressed equally in males and females and confirmed that *Lsp1*α is not dosage compensated. Unlike control X-linked genes, *Lsp1*α was not enriched for H4K16ac in the third instar larval fat body, the tissue in which the gene is actively expressed. X-linked *Lsp1α promoter-lacZ *reporter transgenes are enriched for H4K16ac in third instar larval fat body. An X-linked reporter gene bracketed by *Lsp1*α flanking regions was dosage compensated. One of the genes flanking *Lsp1*α is expressed in the same tissue. This gene shows a modest enrichment for H4K16ac but only at the part of the gene most distant from *Lsp1*α. Phylogenetic analyses of the sequences of the genomes of 12 *Drosophila *species shows that *Lsp1*α is only present within the *melanogaster *subgroup of species.

**Conclusion:**

*Lsp1*α is not modified by the MSL complex but is in a region of the X chromosome that is regulated by the MSL complex. The high activity or tissue-specificity of the *Lsp1*α promoter does not prevent regulation by the MSL complex. The regions flanking *Lsp1*α do not appear to block access by the MSL complex. *Lsp1*α appears to have recently evolved within the *melanogaster *subgroup of *Drosophila *species. The most likely explanation for why *Lsp1*α is not dosage compensated is that the gene has not evolved a mechanism to independently recruit the MSL complex, possibly because of its recent evolutionary origin, and because there appears to be a low level of bound MSL complex in a nearby gene that is active in the same tissue.

## Background

X chromosome dosage compensation in *Drosophila melanogaster *is achieved by doubling the transcription of most genes on the single X chromosome in male flies [1-4]. This dosage compensation is mediated by the male-specific lethal (MSL) complex containing both protein and non-coding RNA components [4]. The genes encoding the MSL proteins were identified through mutagenesis screens, in which the mutant phenotype is male lethality (*male specific lethal *mutations) [5,6]. Five proteins form the core of the MSL complex: MSL1, MSL2, MSL3, MLE and the histone acetyl transferase MOF, which acetylates histone H4 at lysine 16 (H4K16ac) [7,8]. The acetylase activity of MOF is essential for male viability [6]. There is considerable evidence that these proteins associate in a complex that localises specifically to the male X chromosome [9-12]. The male specificity of the complex is due to MSL2, which is negatively regulated at the translational level by the female-specific protein SXL [13,14].

MSL1 and MSL2 are essential and sufficient for binding of a partial complex to ~35 high affinity sites along the X chromosome [9,12,15,16]. Two of these sites correspond to the genes encoding the non-coding RNAs, *roX1 *and *roX2 *(*RNA on the X chromosome*), which are part of the MSL complex [17]. These RNAs are redundant, but essential for dosage compensation, although approximately 5% of male *roX1 roX2 *double mutants survive as adults [18]. It has been proposed that the high affinity binding regions constitute *chromatin entry sites*, at which the MSL complex assembles prior to spreading into flanking regions of chromatin [17]. However, chromatin entry sites are not essential for targeting of the MSL complex [19,20]. An alternative model proposes that the MSL complex is targeted to individual X-linked genes by uncharacterised sequence motifs that are absent from autosomal genes [20]. This model is supported by recent high-resolution chromatin immunoprecipitation studies (ChIP-chip), which found that MSL binding is gene specific [21-23]. However, autosomal genes inserted on the X chromosome can be dosage compensated [24], indicating that bound MSL complex may be able to regulate the expression of nearby genes in the chromatin domain.

The X-linked gene *Larval serum protein 1 alpha *(*Lsp1*α) appears to escape dosage compensation by the MSL complex [25,26]. LSP1α is an abundant protein expressed in the fat body of third instar larvae [27], which forms a complex with autosomal LSP1 proteins to act as nutrient reservoir for pupal development [28]. LSP1α is not essential for survival, as flies carrying mutations in all of the *Lsp1 *genes are viable [29]. *Lsp1*α could escape regulation by the MSL complex by one of two distinct possibilities. Either, it is flanked by boundary elements that block access of the MSL complex or it lacks characteristics required to attract the MSL complex, such as DNA sequences or chromatin composition. Both of these models are supported by the observation that the *Lsp1*α gene is either partially or fully dosage compensated when relocated to two other locations on the male X chromosome [30].

In this study we examine possible explanations for why *Lsp1*α is not dosage compensated.

## Results

### *Lsp1*α is flanked by dosage compensated genes

In order to determine whether *Lsp1*α is the only gene within its chromosomal region to escape dosage compensation, the dosage compensation status of the genes flanking *Lsp1*α was examined. Previous work indicated that the gene 5' of *Lsp1*α, *CG2560*, is dosage compensated [31]. Several other genes in the region near *Lsp1*α were identified after publication of the *Drosophila *genome sequence.

**Figure 1:**
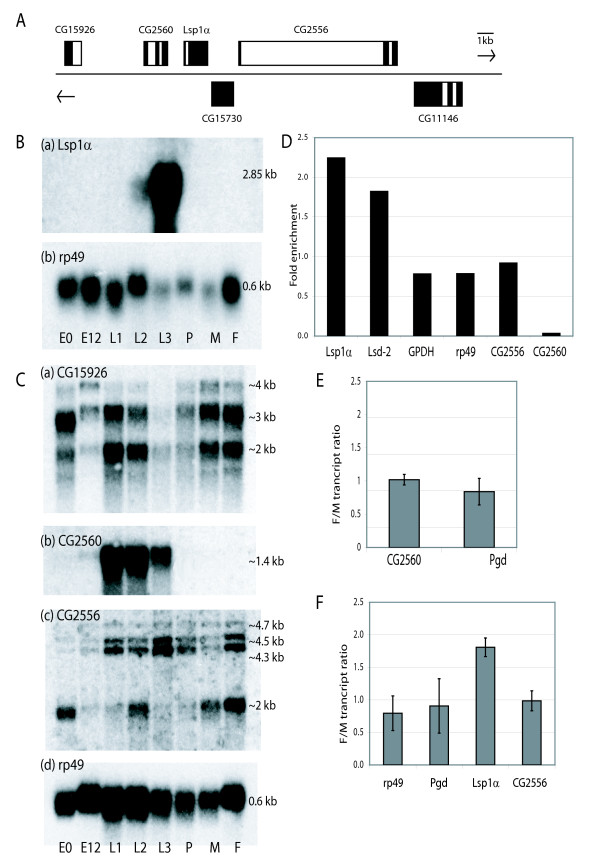
**The genes flanking *Lsp1*α are dosage compensated, and *CG2556 *is expressed in the same tissue as *Lsp1*α **(A) The predicted genes flanking *Lsp1*α (exons in black). (B) Northern RNA hybridization analysis of 10 μg of total RNA from embryos 0 – 2 h after laying (E0), embryos 12 h after laying (E12), first instar larvae (L1), second instar larvae (L2), third instar larvae (L3), pupae (P), adult males (M), and adult females (F). All embryo, larval and pupae samples consist of mixed male and female RNA. Northerns were probed with cDNAs for *Lsp1*α (a) and *rp49 *(b). (C) Northern hybridization analysis of 2 μg of poly(A) mRNA from the developmental stages described in (B). Northerns were probed with cDNAs for *CG15926 *(a), *CG2560 *(b), *CG2556 *(c) and *rp49 *(d). (D) Real-time RT-PCR of *Lsp1*α, *Lsd-2*, *Gpdh*, *rp49*, *CG2556 *and *CG2560 *in male fat body and whole third instar male larvae cDNA. The fold enrichment of each transcript in fat body compared to whole larvae cDNA is shown. (E)*CG2560 *and *Pgd *mRNA was measured by RNase protection relative to *rp49 *in male and female first instar larvae. Mean female/male transcript ratios and 95% confidence intervals are indicated for 3 experiments. (F) *Lsp1*α, *CG2556, Pgd *and *rp49 *mRNA was measured by RNase protection in male and female *y w *staged-third instar larvae. Mean female/male transcript ratios and 95% confidence intervals are indicated for 3 experiments.

Five putative genes exist in the region immediately surrounding *Lsp1*α as predicted by cDNA evidence from the *Drosophila *genome project [32]: *CG15926*, *CG2560 *(referred to as *L12 *by [31]), *CG15730*, *CG2556 *and *CG11146 *(Figure 1A). Since *CG11146 *is separated from *Lsp1*α by two intervening genes, it was not examined in this study. The developmental stage in which *Lsp1*α, *CG15926, CG2560 *and *CG2556 *are expressed was determined by Northern RNA hybridisation analysis (Figure 1B and 1C). As expected *Lsp1*α is very highly expressed and easily detected in total RNA from third instar larvae (Figure 1B). *CG2560 *is expressed in all larval stages, as previously reported. Four transcripts were detected for *CG2556*, which is downstream of *Lsp1*α (Figure 1C). These transcripts are expressed differentially throughout development but importantly are present in third instar larvae. Thus, genes 5' and 3' of *Lsp1*α are expressed at the same stage of development. Transcripts from *CG15730*, the gene immediately 3' of *Lsp1α*, were not detected at any of the developmental stages analysed using 2 μg of poly(A)^+ ^mRNA, or in adults using RT-PCR (data not shown). It is possible that this intron-less gene may be expressed in only a few cells, or may require stimuli not present under standard conditions. Since expression could not be detected, it was not examined further in this study. *CG15926 *transcripts are present in ~2-fold higher levels in hemisected (head plus thorax) adult males compared to females as shown by RNase protection (data not shown), thus the dosage compensation status of this gene could not be determined. There were slightly higher transcript levels of the *rp49 *loading control in whole adult females than males (Figure 1C) as shown previously [33], possibly due to strong ovarian expression. For this reason, hemisected adults or sexed larvae were used in this study for determining a gene's dosage compensation status.

Multi-probe quantitative RNase protection analysis was used to determine the dosage compensation status of *CG2560 *and *CG2556 *genes in first and third instar larvae respectively. Control probes detected transcripts from the X-linked dosage compensated *6-phosphogluconate dehydrogenase *(*Pgd*) [34,35] and constitutive autosomal *ribosomal protein 49 *(*rp49*) [33] genes. First instar larvae were sexed using a stock in which only the male larvae express GFP. Female to male *CG2560 *and *Pgd *transcript ratios were normalised to *rp49*, as the 3 probes were analysed simultaneously. A female to male transcript ratio of one indicates that a gene is compensated, whereas a female to male ratio of two suggests that a gene is not compensated. *CG2560 *and *Pgd *have female to male transcript ratios of 1.02 ± 0.08 and 0.84 ± 0.20 respectively (Figure 1E) indicating that these genes are both dosage compensated. This concurs with the *CG2560 *transcript ratios obtained in second instar larvae using an alternative method [31]. Single probe RNase protection of *CG2556 *and *Lsp1*α was conducted in male and female *y w *staged-third instar larvae relative to *Pgd *and *rp49*, as both transcripts are present at this developmental stage (Figure 1B,C). Female to male *CG2556*, *Lsp1*α and *Pgd *transcript ratios were not normalised to *rp49*, as the 4 probes were analysed separately due to the similar size of the protected RNAs. *Rp49 *and *Pgd *have female to male transcript ratios of 0.79 ± 0.27 and 0.91 ± 0.42 respectively (Figure 1F), demonstrating equivalent RNA levels are present in both sexes. *CG2556 *and *Lsp1*α have female to male transcript ratios of 0.99 ± 0.15 and 1.81 ± 0.14 respectively (Figure 1F), indicating that *CG2556 *is dosage compensated but *Lsp1*α is not. These results show that two of the genes flanking *Lsp1*α are dosage compensated, and suggest that *Lsp1*α is unique within its chromosomal domain in escaping regulation by the MSL complex.

### The regions flanking *Lsp1*α do not contain elements able to block dosage compensation of an X-linked transgene

The genes flanking *Lsp1*α are dosage compensated, but *Lsp1*α is not. One possible explanation for why *Lsp1*α escapes dosage compensation is that flanking sequence elements somehow block access of the MSL complex to the gene. To test this possibility, the genomic regions between *Lsp1*α and *CG2560 *(I) and between *Lsp1*α and *CG2556 *(I2) were inserted either side of an *arm-lacZ *reporter construct (*I-arm-lacZ-I2*). We have previously shown that X-linked *arm-lacZ *transgenes are fully dosage compensated [36,37], although it is not known if this is due to local spreading of the MSL complex or to direct recruitment of the complex. If the *Lsp1*α flanking regions contain elements able to block access of the MSL complex, it follows that X-linked *I-arm-lacZ-I2 *transgenic lines will not be dosage compensated, and will exhibit female to male reporter activity ratios of two. Due to the presence of promoter sequences within the (I) region, female to male reporter activity ratios were analysed in adult flies, in which only the *armadillo *promoter is active, rather than in third instar larvae in which both the *armadillo *and *Lsp1*α promoter sequences are active. All autosomal and X-linked *arm-lacZ *lines had female to male β-galactosidase activity ratios of ~1 (Table 1). Both autosomal *I-arm-lacZ-I2 *lines also had female to male β-galactosidase activity ratios of ~1. The X-linked *I-arm-lacZ-I2 *line exhibited a female to male β-galactosidase activity ratio of ~1, indicating that the (I) and (I2) regions do not contain elements able to prevent the MSL complex from binding to and dosage compensating *arm-lacZ *on the X chromosome.

The precise chromosomal location of these X-linked transgenes was determined by inverse PCR. The *I-arm-lacZ-I2:19C3 *transgene has inserted between the X-linked *CG1631 *and *CG15462 *genes that are uncharacterised with respect to expression and are part of an approx. 140 kb gene poor region of the chromosome (19C2 to 19C5). The nearest genes that showed significant binding of MSL1 and MSL3 in embryos are *Rab10 *(19C1) and *l(1)G0004 *(19C6) that are approximately 95 kb upstream and 45kb downstream respectively from the site of insertion of the *I-arm-lacZ-I2 *transgene [21,23] (Additional file 1; Panel B). The *arm-lacZ:10D8 *transgene has inserted in the first intron of the X-linked *inaF *(*CG2457) *gene, which encodes a protein with calcium channel regulator activity involved in rhodopsin mediated signalling that is expressed in the head and eye of adult flies [38]. Although, it has not been reported if *inaF *is dosage compensated, significant binding of MSL1 is detected at the 3' end of this gene in embryos [23].

### *Lsp1*α is not enriched for histone H4 acetylated at lysine 16 in male larval fat body nuclei

**Figure 2:**
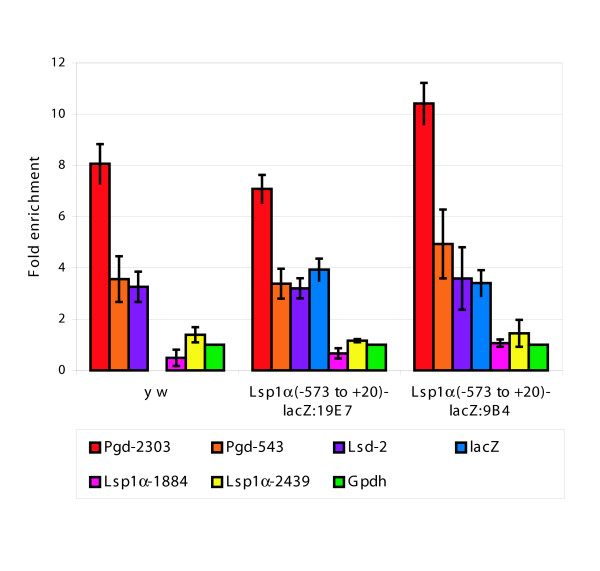
***Lsp1*α is not acetylated at H4K16, but X-linked *Lsp1α (-573 to +20)-lacZ *transgenes are**. Chromatin from *y w*, *Lsp1α (-573 to +20)-lacZ:19E7 *and *Lsp1α (-573 to +20)-lacZ:9B4 *male third instar larval fat body nuclei immunoprecipitated with antibody against H4K16ac. The fold enrichment of immunoprecipitated DNA relative to input DNA is shown for two experiments (95% confidence intervals indicated). Fold enrichment is normalized to *Gpdh*, which is set to 1. A 3 – 10 fold enrichment is observed for the control genes *Pgd *and *Lsd-2 *and both X-linked *Lsp1α (-573 to +20)-lacZ *transgenes. However, no enrichment is observed for *Lsp1*α. Two primer sets were used to amplify different regions within the *Pgd *and *Lsp1*α genes. All primers are designed to the 3' UTR or 3' region of the open reading frame with the exception of the *Pgd*-543 set, which is within the second intron but towards the 5' end of the *Pgd *gene.

There was no binding of MSL1 or MSL3 to *Lsp1*α in embryos or of MSL3 to *Lsp1*α in SL2 cells [21-23] (Additional file 1; Panel D). This would be the expected result since *Lsp1*α is not dosage compensated. In SL2 cells, ~90% of MSL3 binding clusters were within expressed genes, with an enrichment in the middle and 3' end [21]. Since *Lsp1*α is not actively expressed in SL2 cells it was possible that MSL complex could be binding to *Lsp1*α in the tissue in which the gene is expressed, namely third instar larval fat body. As acetylation of H4 at lysine 16 is dependent on the MOF component of the MSL complex, we measured the relative level of H4K16ac on *Lsp1*α in larval fat body nuclei by chromatin immunoprecipitation analysis. Chromatin from hand-dissected male *y w *larval fat body was immunoprecipitated with antibody against H4K16ac. The X-linked, fat body-expressed *Lipid storage droplet-2 *(*Lsd-2*) and *Pgd *genes showed 3 – 10 fold enrichments after immunoprecipitation compared to the autosomal gene, *Glycerol-3-phosphate dehydrogenase (Gpdh)*, which had a relative enrichment of one (Figure 2). The differential levels of enrichment within *Pgd *have been observed previously [39]. *Lsp1*α exhibited no enrichment for H4K16ac, confirming the prediction that actively expressed *Lsp1*α would not be acetylated by MOF as it is not dosage compensated.

### *Lsp1*α promoter-*lacZ *X-linked transgenes are enriched for H4K16ac in larval fat body

Since the majority of MSL target genes are widely expressed [21-23], we next investigated if the *Lsp1*α gene was not enriched for H4K16ac because of the high activity and tissue specificity of its promoter. The *Lsp1*α gene promoter was fused to the *lacZ *reporter gene and two X-linked lines containing this gene construct were obtained. Chromatin from male *Lsp1α (-573 to +20)-lacZ:9B4 *and *Lsp1α (-573 to +20)-lacZ:19E7 *third instar larval fat bodies was immunoprecipitated with antibody against H4K16ac (Figure 2). As for the *y w *strain, the X-linked *Lsd-2 *and *Pgd *genes were enriched for H4K16ac. The two X-linked *Lsp1α (-573 to +20)-lacZ *transgenes also showed 3-fold enrichments within *lacZ*. Thus the lack of enrichment of H4K16ac within *Lsp1*α is not because of the tissue-specificity of the gene promoter.

There are two possible explanations for why X-linked *Lsp1α-lacZ *transgenes were enriched for H4K16ac in larval fat body. Either the transgenes have inserted near MSL complex target genes or alternatively MSL complex is directly recruited to the *lacZ *gene. The latter would seem less likely as the gene is of bacterial origin and MSL complex is not recruited to autosomally integrated *lacZ *transgenes [37]. The *Lsp1α (-573 to +20)-lacZ:9B4 *transgene has inserted in the first intron of the *CG15309 *gene. While there are no MSL1 or MSL3 binding sites within *CG15309*, there are clusters of sites within the closely adjacent *l(1)G0230 *gene in embryos, SL2 and Clone 8 cells (Additional file 1; Panel C). The *Lsp1α (-573 to +20)-lacZ:19E7 *transgene has inserted between the *CG1529 *and *Ntf-2 *(*CG1740*) genes. There are clusters of MSL1 and MSL3 binding sites within both the *CG1529 *and *Ntf-2 *genes in embryos, SL2 and Clone 8 cells [21,23] (Additional file 1; Panel A). While it is not known if *CG15309*, *CG1529 *or *Ntf-2 *gene are actively expressed and targeted by the MSL complex in third instar larval fat body, *Ntf-2 *is likely to be expressed in this tissue based on mutant phenotypes [40]. That MSL complex distribution appears to remain largely stable across development [21,22] would suggest that MSL complex would be bound to the *CG15309*, *CG1529 *and *Ntf-2 *genes in larval fat body.

### *CG2556 *is expressed in larval fat body but shows only a moderate enrichment for H4K16ac at the 3' end

If X-linked *Lsp1α-lacZ *transgenes are enriched for H4K16ac because they have inserted near MSL target genes then why is *Lsp1*α not enriched for H4K16ac as the flanking genes are dosage compensated? Since there is a strong correlation between MSL complex binding and gene transcription [21], one possibility is that the flanking genes are not transcribed in third instar larval fat body. Both *CG2560 *and *CG2556 *are expressed in third instar larvae (Figure 1C), but their tissue distribution is unknown. In order to determine whether *CG2560 *and *CG2556 *are expressed in the fat body, real-time RT-PCR of cDNA from male fat body and whole larvae was conducted on *CG2560 *and *CG2556 *relative to the fat body specific genes *Lsp1*α [27] and *Lsd-2 *[41], and the constitutively expressed genes *Gpdh *and *rp49*. *Lsp1*α and *Lsd-2 *show 2.24 and 1.82-fold enrichments respectively in fat body cDNA compared to whole third instar larval cDNA, while *Gpdh *and *rp49 *show 0.78 and 0.79-fold enrichments respectively (Figure 1D). *CG2560 *demonstrates 0.03-fold enrichment in fat body cDNA compared to whole third instar larval cDNA, indicating that it is not expressed in fat body tissue. This is consistent with its proposed function as a structural component of the larval cuticle [42] and specific expression in dorsal and ventral epidermis in late embryos [43]. *CG2556 *shows 0.92-fold enrichment in fat body cDNA compared to whole third instar larval cDNA, indicating that it is expressed in both this tissue and other parts of the larvae. Transcripts for the gene immediately 3' of *Lsp1*α, *CG15730*, were not detected in third instar larvae hence it is unlikely that the MSL complex is targeted to this gene in fat body tissue.

Since *CG2556 *is also expressed in fat body we performed ChIP experiments with isolated fat body nuclei and anti-H4K16ac antibody. There was no enrichment with a 5' UTR fragment (fold enrichment of 1.10 ± 0.09 relative to *Gpdh *in *y w *male fat bodies). Further, the 3' UTR of *CG2556 *is only moderately enriched for this histone modification (fold enrichment of 1.93 ± 0.77 relative to *Gpdh *in *y w *male fat bodies), suggesting MSL complex is not present in high levels in this region of the chromosome in third instar larval fat body, although the gene is clearly dosage compensated. The moderate enrichment of H4K16ac at the 3' end of *CG2556 *is consistent with the high-density ChIP-chip profiles that found that the MSL complex binds to intragenic regions, particularly the 3' end of X-linked genes [21,23].

### *Lsp1*α has most likely evolved recently within the *melanogaster *subgroup of *Drosophila *species

**Figure 3:**
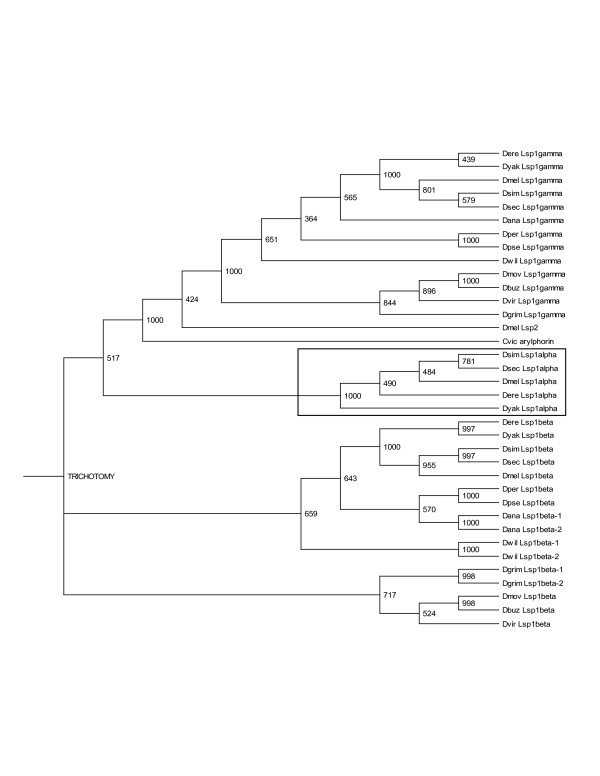
***Lsp1*α is present in five closely related *Drosophila *species, but is absent in more distantly related species**. Maximum likelihood tree based on the ungapped regions of a ClustalX alignment (additional file 4) of the LSP1α, LSP1β and LSP1γ protein sequences from *D. melanogaster *(Dmel), *D. yakuba *(Dyak), *D. erecta *(Dere), *D. simulans *(Dsim), *D. sechellia *(Dsec), *D. mojavensis (*Dmoj), *D. buzzatii *(Dbuz), *D. ananassae *(Dana), *D. pseudoobscura *(Dpse), *D. willistoni *(Dwil), *D. grimshawi *(Dgri), *D. persimilis *(Dper) and *D. virilis *(Dvir). The Lsp1-like protein arylphorin from the blowfly *Calliphora vicina *[61] is also included and bootstrap support is shown on the branches. LSP1α homologues (in the boxed region) are present only in *D. melanogaster*, *D. yakuba*, *D. erecta*, *D. simulans *and *D. sechellia*.

It had been suggested that *Lsp1*α has arisen from a duplication and translocation of an autosomal *Lsp1*β gene [44]. The sequencing of the genomes of twelve *Drosophila *species [45] allowed us to address when *Lsp1*α evolved. As expected homologues of *Lsp1*β and *Lsp1γ *were found in all *Drosophila *species examined. *Lsp1*α homologues, however, are only present in the *melanogaster *subgroup of species, which are thought to have descended from a common ancestor 8 – 12 million years ago [46] (Figure 3). In these species *Lsp1*α lies between homologues of *CG2560 *and *CG15730*, while in the remainder of the species these genes are immediately adjacent (including *D. ananassae*, which is part of the *melanogaster *group but not sub-group). Thus it would appear that *Lsp1*α arose relatively recently within the *melanogaster *subgroup of species and so may not have yet evolved MSL binding sites. However, a tree based on maximum likelihood analysis of LSP1 sequences (Methods) suggests that *Lsp1*α arose before the divergence of *Drosophila *species (Figure 3). If so, then *Lsp1*α has been precisely lost on at least four separate occasions (ancestor of: *D. ananassae*, *obscura *group, *willistoni *group and *Drosophila *subgenus). More likely some residues within LSP1α proteins may not be under the same functional constraints as in LSP1β proteins leading to the observed divergence. The *Lsp1*β gene seems to be particularly prone to duplication events as duplicated *Lsp1*β genes were found in *D. ananassae*, *D. grimshawi *and *D. willistoni *genome sequences (Figure 3). The two *Lsp1*β genes are immediately adjacent to each other in these three species. The maximum likelihood analysis suggests the duplication events have occurred recently within each of the three species.

## Discussion

*Lsp1*α is a well characterised example of a non-dosage compensated gene [25]. In contrast, two genes situated less than 5 kb either side of it, are expressed equivalently in male and female larvae. That *Lsp1*α escapes regulation by the MSL complex was shown by the lack of enrichment for H4K16ac in the tissue in which it is expressed. These results are consistent with high-resolution ChIP-chip studies that found that MSL complex binding was gene-specific [21-23]. Further, the complex bound predominantly to constitutively expressed genes. *Lsp1*α is certainly not a housekeeping gene, but rather is a gene that is very highly expressed in a specific cell type and a specific stage of development. Since *Lsp1*α is not an essential gene [29] there would have been little evolutionary pressure to acquire MSL binding sites since it evolved, which appears to have been relatively recently in the *melanogaster *subgroup of species. The only gene in the *Lsp1*α gene region that is bound to MSL1 and MSL3 in embryos is *Rab40 *[21,23] (Additional file 1), which is approx. 30 kb upstream of *Lsp1*α. Thus *Lsp1*α appears to have arisen within a region of the X chromosome that has few strong MSL binding sites in whole embryos.

In the two-step model for MSL complex targeting to X chromosome genes, the complex is initially bound to sequences of higher affinity and then spreads locally to nearby expressed genes [17]. Such a mechanism would explain why autosomal genes inserted onto the X chromosome can be dosage compensated [24]. According to this model, it would be anticipated that the MSL complex could spread locally from flanking dosage compensated genes that are active in third instar larval fat cells to *Lsp1*α. Of the two flanking dosage compensated genes, only the downstream *CG2556 *gene is transcribed in third instar larval fat cells. However, we could not detect any enrichment for H4K16ac at the 5' end and only a modest enrichment for H4K16ac at the 3' end, which is ~11 kb from *Lsp1*α. Thus it appears that MSL complex does not spread to the *Lsp1*α gene in its normal chromatin location because the level of complex bound to nearby active genes in fat body nuclei is low. The ChIP-chip studies identified several examples of neighbouring genes that have differential MSL binding profiles. It remains to be determined if, like *Lsp1*α, the unbound genes are not enriched for H4K16ac in the cells in which they are actively transcribed. If so, it would be of interest to know if these genes, like *Lsp1*α [30], can respond to the MSL complex when relocated to other locations on the X chromosome.

We found no evidence for boundary elements flanking the *Lsp1*α gene that could prevent access of the MSL complex to an active gene. An *arm-lacZ *reporter gene bracketed by sequences that flank the *Lsp1*α gene was fully dosage compensated when inserted onto the X chromosome. The MSL complex could reach the *I-arm-lacZ-I2 *transgene by spreading from a nearby gene with bound complex or could bind directly to the transgene. The *I-arm-lacZ-I2 *transgene inserted into a very gene poor region that is largely devoid of bound MSL complex in embryos and SL2 cells [21,23]. The nearest gene with significant levels of bound complex in embryos is approx. 45 kb from the transgene insertion site. The MSL complex can spread hundreds of kilobases from an autosomally integrated *roX *gene [17], so it is possible that the MSL complex could spread 45kb along the male X chromosome. It is also possible that MSL complex may be bound to more genes in this region in adults, the stage we measured β-galactosidase activity. However, while MSL complex binding pattern is not invariant, it is largely similar in distinct cell types [21,22]. Thus, while it is clear that *Lsp1*α is not flanked by sequences that prevent access of the MSL complex, we cannot conclude if this is because they fail to block local spreading of the complex. If MSL complex does not spread locally to the integrated *arm-lacZ *reporter gene, then the transgene must independently recruit MSL complex. This may simply be because the MSL complex preferentially binds to expressed genes [21]. However, transcription is not sufficient to recruit complex. Legube *et al *(2006) [22] found that the promoter regions of some MSL1 target genes are enriched in DREF binding sites. The *arm *promoter has several possible DREF binding sites (not shown). *armadillo *is an X-linked constitutively expressed gene [47]. The 1.6 kb *arm *fragment in the *arm-lacZ *construct contains 5' flanking sequence, the two major start sites of transcription, the first intron and start of second exon [47]. There is significant binding of MSL1 and MSL3 to this fragment of the *arm *gene in embryos and SL2 cells respectively [21,23]. Thus the *arm *promoter may contribute to recruitment of MSL complex to an actively expressed *arm-lacZ *transgene. While *arm-lacZ *transgenes are fully dosage compensated at several locations on the X chromosome [36,37], there is no binding of the MSL complex to autosomally integrated *arm-lacZ *transgenes, which are equally expressed in males and females [37]. Thus if the *arm-lacZ *transgene can independently recruit MSL complex, it can only do so in an X chromosomal environment. Clearly additional studies are needed to identify what features of the *arm-lacZ *transgene are important for recruitment of the MSL complex. The development of site-specific integration systems for *Drosophila *[48,49] should greatly facilitate such studies as various gene constructs could all be tested at the same locations on the X chromosome.

## Conclusion

In this study we have examined possible explanations for why the X-lined *Lsp1*α gene is not dosage compensated. *Lsp1*α is not enriched for H4K16ac in third instar larval fat body, the tissue in which the gene is actively expressed. Thus *Lsp1*α is not compensated because the chromatin is not modified by the MSL complex at the gene's normal location on the X chromosome. *Lsp1*α is in a region of the X chromosome that is subject to regulation by the MSL complex, as genes flanking and within 5 kb of *Lsp1*α are dosage compensated. *Lsp1*α does not appear to be surrounded by sequence elements that prevent access of the MSL complex as these flanking regions did not prevent a reporter gene from being dosage compensated when inserted on the X chromosome. The stage-specificity or high activity of the *Lsp1*α promoter does not prevent dosage compensation because X-linked *lacZ *transgenes under the control of the *Lsp1*α promoter were enriched for H4K16ac in larval fat body. Only one of the genes flanking *Lsp1*α is expressed in the same tissue as *Lsp1*α and this gene showed no enrichment for H4K16ac at its 5' end (end closest to *Lsp1*α) and showed only a modest enrichment at its 3' end in larval fat body. Homologues of *Lsp1*α were found only in the *melanogaster *subgroup of species. The most likely explanation for why *Lsp1*α is not dosage compensated is that the gene has not evolved a mechanism to independently recruit the MSL complex, possibly because of its recent evolutionary origin, and because there appears to be a low level of bound MSL complex in a nearby gene that is active in the same tissue.

Recent ChIP-chip analyses have identified several expressed X-linked genes that are not bound by the MSL complex. The significance of this study is that we have addressed possible mechanisms by which one such gene escapes regulation by the MSL complex.

## Methods

### Northern RNA Hybridisation Analysis

RNA was extracted using TRIzol reagent (Invitrogen) and RNA *secure *(Ambion). Poly(A) RNA was isolated using oligo(dT) cellulose (Roche) and Northern hybridization analysis conducted as described in [50].

### Real-time RT-PCR Assays

RNA from 10 male third instar larvae or 10 male third instar larval fat bodies was treated with Turbo DNase (Ambion) and reverse transcribed (Roche). Quantitative real-time PCR was conducted in triplicate (variation <15%) using the LightCycler FastStart DNA Master^PLUS ^SYBR Green I reaction mix (Roche) in a LightCycler Instrument (Roche) on 2 μl of 10-fold or 100-fold diluted cDNA in a 10 μl reaction volume. Information about the primers used in this study is available upon request. An annealing temperature of 55°C and an extension of 12 s were used. The crossing point (CP) was automatically determined by the LightCycler software (Roche). Fold enrichment was determined by 2^^^(CP whole larvae – CP fat body). The primer sets used were; *Lsp1*α (5'CTCGCTGACGGACAAC and 5'GGGCTCAGTAAGGTCCA), *Rp49 *(5'CGGTTACGGATCGAACA and 5'CGATCTCGCCGCAGTAAA), *Lsd-2 *(5'AGTGTACTAGCCGATACG and 5'TCTGACTCCCGGATCT), *CG2560 *(5'ATGGCAATGCTTACGGT and 5'GAGGTGGCTGATAATCGTAG), *CG2556 *(5'TGGTAATGGCGGCCTAAA and 5'TGCGAGTGTTCAGCTTG), *Gpdh *(5'GTGCCCGACCTGGTTGAG and 5'CTTGCCTTCAGGTGACGC).

### Quantitative RNase Protection Assays

Quantitative RNase protection was conducted on 3 – 4 separate collections of matched female and male *FM7I, P{w [+mC]=Act GFP}JMR3/c(1)DX,y^1^f^1 ^*first instar larvae or *y w *blue food-staged third instar larvae [51] using the RPA III kit (Ambion). Antisense RNA probes for *CG2560*, *Pgd*, *rp49*, *Lsp1*α and *CG2556 *were synthesized with T7, T3 or SP6 RNA polymerase (Roche). The relative radioactivity of the probes was adjusted by increasing the concentration of [α-^32^P]CTP and decreasing the concentration of unlabeled CTP in the reaction cocktails. Unincorporated radionucleotide was removed with the NucAway spin column (Ambion). The *CG2556 *probe was gel purified (Qiagen). 3 – 10 fold molar excess of probe was added to 4 μg (*CG2560*, *Pgd*, *rp49 *and *Lsp1*α) or 20 μg (*CG2556*) of DNase-treated phenol/chloroform purified RNA and annealed overnight with RNA in the presence of pellet paint co-precipitant (Novagen); protected RNA probes were detected and quantified on 5% polyacrylamide urea gels with the Storm 860 phosphorimager (Molecular Dynamics) or the FLA-5000 phosphorimager (Fujifilm). The quantification value of each protected RNA species was corrected for the background value of the sample of yeast RNA hybridized to probe treated with RNase. The mass of RNA used in each assay was determined to be within the linear range of the RNase protection assay for each protected RNA. The sizes of the protected RNAs were 366 nt for *CG2560*, 391 nt + 294 nt for *Lsp1*α, 268 nt for *CG2556*, 171 nt + 43 nt for *Pgd *and 312 nt for *rp49*.

### Generation of Transgenic Fly Lines

All recombinant DNA manipulations were carried out using standard procedures [50] unless otherwise specified. The 883 bp region between *Lsp1*α and *CG2560 *including the *Lsp1*α promoter (I) and 4596 bp region between the 3' end of *Lsp1*α and the 5' end of *CG2556 *(I2) were amplified by PCR from genomic *y *w *D. melanogaster *DNA, and cloned into pGEM-T Easy (Promega). The primer sequences used are available upon request. *Pst*I-*Not*I and *EcoR*I-*Stu*I linkers were inserted into the *Pst*I site 3' of *lacZ-SV40 *and the *EcoR*I site 5' of *armadillo *in pCaSpeR-arm-βgal (*arm-lacZ*) [47]. The blunt-ended *Not*I (I) and *Not*I (I2) fragments were cloned into the *Stu*I and *Not*I sites of this plasmid respectively, generating *I-arm-lacZ-I2*. The 593 bp *Lsp1*α promoter (-573 to +20) was amplified by PCR from genomic *y *w *D. melanogaster *DNA, and cloned into pGEM-T Easy (Promega). The blunt-ended *Not*I promoter fragment was cloned into the *Stu*I site of pCaSpeR-arm-βgal in which the *EcoR*I/*Asp*718 *armadillo *promoter fragment had been replaced with a linker containing *EcoR*I,*BamH*I, *Nhe*I, *Stu*I and *Asp*718 sites, generating the *Lsp1α (-573 to +20)-lacZ *construct. Transgenic flies carrying these constructs were generated from *y w *stock using standard procedures [52]. The site of transgene integration was determined where possible using inverse PCR, and all transgenic lines consist of single insertions as determined by Southern hybridisation analysis.

### β-galactosidase Assays

β-galactosidase assays were performed on hemisected adults as described in [36]. Assays were performed in triplicate on 3 separate collections unless otherwise stated. Means and standard deviations of activities and ratios were calculated from the 3 separate collections.

### Chromatin Immunoprecipitation Assays

Chromatin immunoprecipitation was based on the procedures described in [53-55] with additional modifications suggested by Edwin Smith (Emory University, 2005, personal communications). Fat bodies manually dissected from 200 male third instar larvae were quick frozen then ground in liquid nitrogen, and homogenized in 5 ml of 10 mM Hepes [pH 7.6], 1 mM EDTA, 150 mM NaCl, 0.6% Triton X-100, 4 mM DTT, 10 mM sodium butyrate with protease inhibitors (Roche). After centrifugation at 500 × *g *for 30 s at 4°C, the supernatant was stored on ice for 5 min, followed by centrifugation at 1500 × *g *for 10 min at 4°C. Pelleted nuclei were resuspended in 360 μl of nucleus isolation buffer (NIB: 0.25 M sucrose, 10 mM Tris-HCl [pH 7.4], 3 mM CaCl_2_, 10 mM sodium butyrate, protease inhibitors) and incubated with formaldehyde at a final concentration of 1% for 10 min at room temperature with shaking. Nuclei were pelleted at 1500 × *g *for 10 min at 4°C, resuspended in 360 μl of NIB, and pelleted. Nuclei were resuspended in 200 μl of 1% SDS, 50 mM Tris-HCl [pH 8.1], 10 mM EDTA, 10 mM sodium butyrate with protease inhibitors for 10 min on ice, followed by sonication in the presence of 425–600 mm acid-washed glass beads (Sigma) for 6 × 30 s pulses at power level 1.5 (VirSonic). This sonication produced DNA fragments with a mean size of 500 bp. The sonicated chromatin lysate was diluted with 1.8 ml of chromatin immunoprecipitation buffer (CIB: 25 mM Tris-HCl [pH 8.0], 137 mM NaCl, 2.7 mM KCl, 1% Triton X-100, 1 mM EDTA, 10 mM sodium butyrate) and centrifuged at 14,000 × *g *for 10 min at 4°C. Input DNA was purified from 200 μl of this supernatant by incubation with 10 μl 10 mg/ml RNase for 10 min at 37°C, and 20 μl 10 mg/ml proteinase K for 6 h at 37°C. Crosslinks were reversed by incubation for 6 h at 65°C. DNA was purified using the QIAquick PCR purification kit (Qiagen).

The remaining 1.8 ml of sonicated chromatin lysate was pre-incubated with 60 μl 50% Protein A Sepharose Bead suspension (Sigma), protease inhibitors and 9 μl 10 mg/ml salmon sperm DNA (ssDNA) for 1 h with shaking at 4°C. 6 μl of rabbit anti histone H4 (Ac16) antibody (AHP417; Serotec) was pre-bound to 120 μl 50% Protein A Sepharose Beads suspension in 1 ml of CIB with protease inhibitors, 10 μl 10 mg/ml ssDNA and 20 μl 10 mg/ml BSA (NEB) for 1 h with shaking at 4°C. The pre-cleared chromatin lysate was incubated with the pre-bound antibody-beads for 3 h at 4°C with shaking. Following this the beads were washed successively in: 150 mM NaCl, 20 mM Tris-HCl [pH 8.0], 2 mM EDTA, 1% Triton X-100, 0.1% SDS, 10 mM sodium butyrate; 500 mM NaCl, 20 mM Tris-HCl [pH 8.0], 2 mM EDTA, 1% Triton X-100, 0.1% SDS, 10 mM sodium butyrate; 250 mM LiCl, 10 mM Tris-HCl [pH 8.0], 1 mM EDTA, 1% sodium deoxycholate, 1% IGEPAL CA-630 (Sigma), 10 mM sodium butyrate; TE buffer. Beads were rinsed and suspended in TE Buffer. Immunoprecipitated DNA (ChIP DNA) was incubated with 1 μl 10 mg/ml RNase for 10 min at 37°C, followed by incubation with 5 μl 10% SDS and 2 μl 10 mg/ml proteinase K for 6 h at 37°C. Crosslink reversal and DNA purification were conducted as described for input DNA.

Quantitative real-time PCR was conducted as described for real-time RT-PCR assays. Undiluted immunoprecipitated DNA (2 μl) and 100-fold diluted input DNA (2 μl) were assayed with each primer set in triplicate (variation <15%). The primer pairs used were; *Lsp1*α-1184 (5'CTCGCTGACGGACAAC and 5'GGGCTCAGTAAGGTCCA), *Lsp1*α-2439 (5'CTTCAAGTTGTAGATCTGATAACATTCCGA and 5'GCTTAACAGAGAAATCTGAATACGTTGG), *Pgd*-543 (5'GGATAAGCAGGTGGAAGTAGGAAG and 5'ACACTTGTGGTTACGGTTTTCG), *Pgd*-2303 (5'GAAGGGCACGGGCAAGTG and 5'CAATGCCGCCGTAATTAAGTCTC), *Lsd-2 *(5'GAAACACACGCACACGG and 5'TCCCAGCGAGCGTACAA), *Gpdh *(5'GTGCCCGACCTGGTTGAG and 5'CTTGCCTTCAGGTGACGC), *lacZ *(5'GCGCGAATTGAATTATGGCCC and 5'GCCATGTGCCTTCTTCCG). The *Pgd *and *Gpdh *primer sets are identical to those used in a previous study [39]. The identity of the PCR products amplified by each primer combination was confirmed by sequencing. Fold enrichment was determined by 2°CP input *X-linked gene *– CP ChIP *X-linked gene*)/2°CP *Gpdh *– CP ChIP *Gpdh*).

### Computational Identification and Analysis of LSP1 Proteins from *Drosophila *species

The *Drosophila melanogaster *LSP1α (GenBank accession no. NP_511138), LSP1β (GenBank accession no. NP_476624) and LSP1γ (GenBank accession no. NP_523868) protein sequences were used to search the nucleotide sequences available in "DroSpeGe: Drosophila Species Genomes" [45] using tBlastn . The scaffolds on which the LSP1 homologues were identified are described in additional files 2 and 3. LSP1β and LSP1γ had independently been identified in *D. buzzatii *and *D. pseudoobscura *[44], as had LSP1γ from *D. simulans *(GenBank accession no. AAB71667). An alignment of the dataset was performed in ClustalX [56], ambiguous bases and gaps were removed from the alignment using PAUP*4.10b [57]. Using ProtTest [58], the optimal model of sequence evolution was determined to be WaG [59]. A maximum likelihood analysis was performed on the data in Phyml using the WaG model with 100 bootstrap replicates [60]. The *D. simulans *LSP1α sequence is incomplete due to a gap in the genomic sequence.

## Authors' contributions

VW carried out all of the experiments and drafted the manuscript. MS conceived of the study, and participated in its design and coordination and helped to draft the manuscript. All authors read and approved the final manuscript.

## Supplementary Material

Additional file 1 **High resolution ChIP-chip profiles of MSL complex binding in the *Lsp1*α, *CG15309*, *CG1529 *and *CG1631 *gene regions**. Summary of data from Gilfillan *et al *(2006) [23] and Alekseyenko *et al *(2006) [21]. The figures were downloaded from the web site maintained by the Becker group [62]. The *Lsp1*α*(-573 to +20)-lacZ:19E7 *insertion site (A), *I-arm-lacZ-I2:19C3 *insertion site (B) and *Lsp1*α*(-573 to +20)-lacZ:9B4 *insertion site (C) are shown in comparison to the *Lsp1*α genomic position at 11A12 (D). Click here for file

Additional file 2 *Lsp1*α is flanked by *CG2560 *and *CG15730 *in five *Drosophila *species, but these genes lie immediately adjacent to one another in the other species that lack *Lsp1*α Click here for file

Additional file 3 *Lsp1 *gene identification in *Drosophila *species. Click here for file

Additional file 4 Clustal X alignment of *Drosophila *Lsp1 sequences and Lsp1-like protein arylphorin from *C. vicina*. Click here for file

## Figures and Tables

**Table 1:** Mean male and female β-galactosidase activities and ratios in X-linked and autosomal *l-arm-lacz-l2 *and *arm-lacZ *adults.

Construct	location	Dose		n^a^	Mean female/male ratio of activity^b^	Mean male activity/copy	Mean female activity/copy
		M	F				
arm-lacZ	68D3 (A)	1	1	3	0.99 ± 0.09^c^	2.36 ± 0.13	2.33 ± 0.09
arm-lacZ	79A4 (A)	2	2	3	0.92 ± 0.09	2.61 ± 0.16	2.39 ± 0.12
arm-lacZ	10D8 (X)	1	2	3	1.12 ± 0.26	4.27 ± 0.51	2.36 ± 0.27
I-arm-lacZ-I2	21A2 (A)	2	2	3	1.00 ± 0.05	2.46 ± 0.43	2.45 ± 0.35
I-arm-lacZ-I2	57A6 (A)	2	2	3	0.99 ± 0.07	2.62 ± 0.20	2.58 ± 0.13
I-arm-lacZ-I2	19C3 (X)	1	2	3	1.11 ± 0.06	4.02 ± 0.27	2.22 ± 0.12

^a ^Number of independent experiments

^b ^100mOD min^-1 ^mg protein

^c ^95% confidence intervals are indicated.
