# Supplementary figures and images for: The non-dosage compensated Lsp1α gene of Drosophila melanogaster escapes acetylation by MOF in larval fat body nuclei, but is flanked by two dosage compensated genes
Source: BMC Mol Biol. 2007 May 19;8:35. doi: 10.1186/1471-2199-8-35 (PMC1890558; doi:10.1186/1471-2199-8-35)

**A**

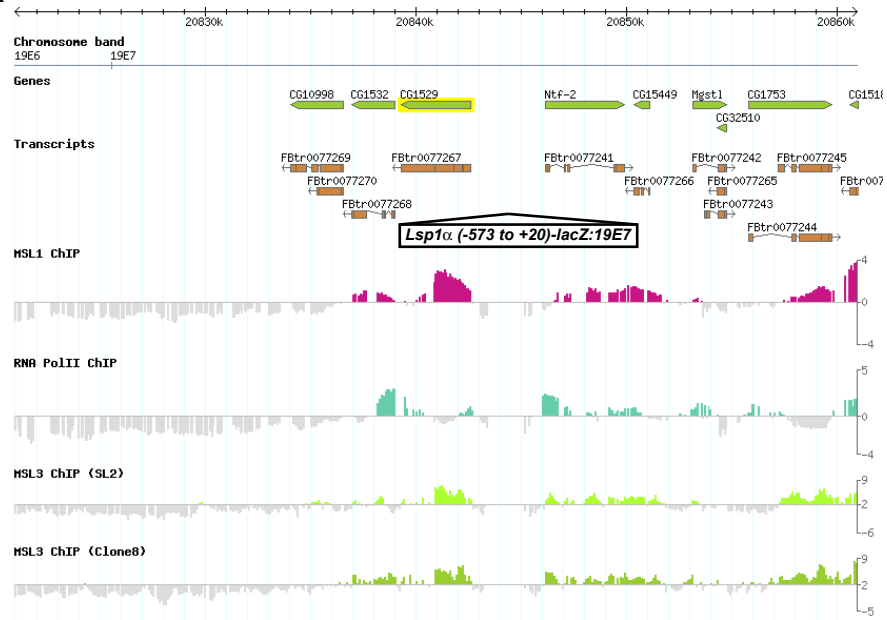

**C**

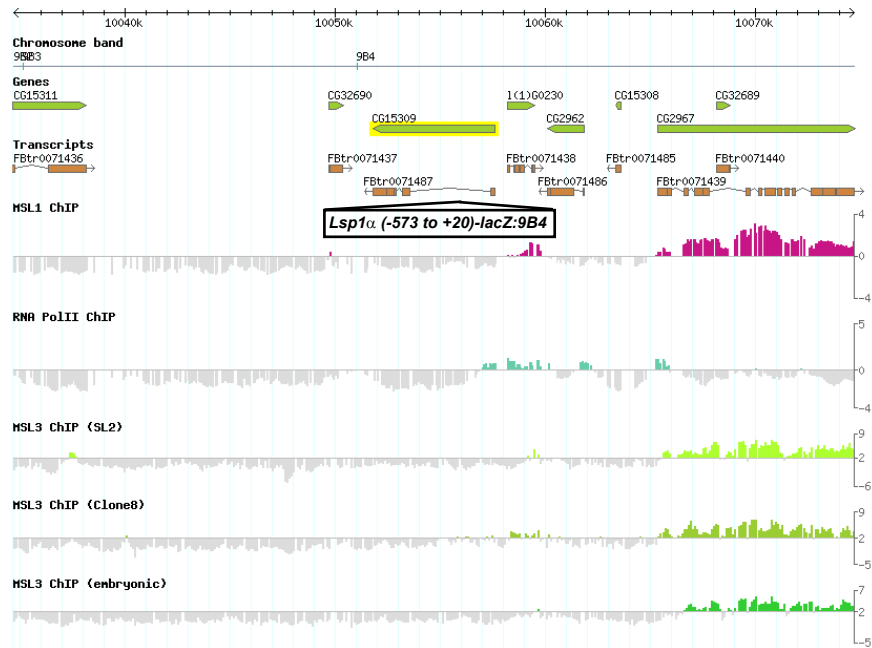**B**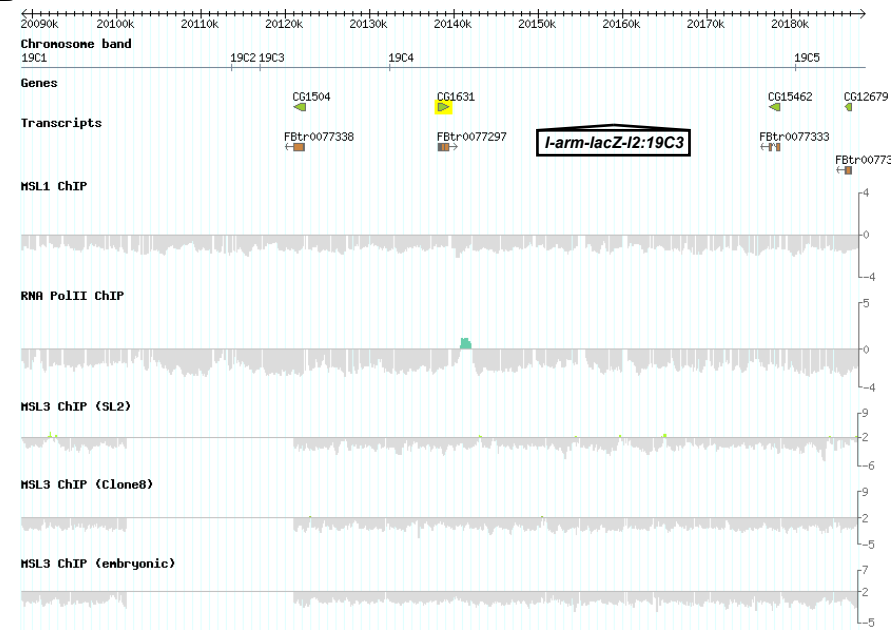

D

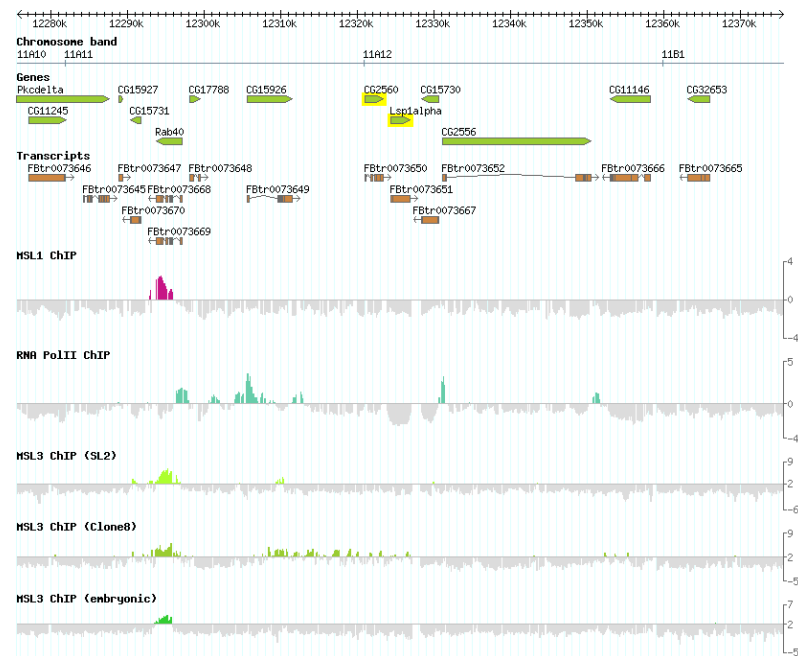

Supplement: Additional file 1 — High resolution ChIP-chip profiles of MSL complex binding in the Lsp1α, CG15309, CG1529 and CG1631 gene regions. Summary of data from Gilfillan et al (2006) [23] and Alekseyenko et al (2006) [21]. The figures were downloaded from the web site maintained by the Becker group [62]. The Lsp1α(-573 to +20)-lacZ:19E7 insertion site (A), I-arm-lacZ-I2:19C3 insertion site (B) and Lsp1α(-573 to +20)-lacZ:9B4 insertion site (C) are shown in comparison to the Lsp1α genomic position at 11A12 (D). [file 1471-2199-8-35-S1.pdf]
