# Supplementary material for: The non-dosage compensated Lsp1α gene of Drosophila melanogaster escapes acetylation by MOF in larval fat body nuclei, but is flanked by two dosage compensated genes
Source: BMC Mol Biol. 2007 May 19;8:35. doi: 10.1186/1471-2199-8-35 (PMC1890558; doi:10.1186/1471-2199-8-35)
Supplement: Additional file 2 — Lsp1α is flanked by CG2560 and CG15730 in five Drosophila species, but these genes lie immediately adjacent to one another in the other species that lack Lsp1α [file 1471-2199-8-35-S2.doc]

**Additional file 2. *Lsp1α* is flanked by *CG2560* and *CG15730* in five *Drosophila* species, but these genes lie immediately adjacent to one another in the species that lack *Lsp1α***

| Species | Gene location | | | | |
| --- | --- | --- | --- | --- | --- |
|  | *CG2560* | *Lsp1a* | *CG15730* | *Lsp1β* | *Lsp1γ* |
| *D. melanogaster* | X:11A7-9 | X:11A12 | X:11A12 | 2L:21E2 | 3L:61A6 |
| *D. erecta* | scaffold_4690:  14474452..14476074 | scaffold_4690:  14470829..14473311 | scaffold_4690:  14470528..14468465 | scaffold_4929:  959327..956878 | scaffold_4784:  39369..36985 |
| *D. yakuba* | X:  6658231..6656337 | X:  6663672..6661165 | X:  6663902..6665968 | 2L:  886148..883701 | 3L:  35009..32633 |
| *D. simulans* | X:  9903604..9906505 | X:  9907431-9910040 | X:  913335-9911742 | 2L:  914510..917073 | 3L:  45579..47953 |
| *D. sechellia* | scaffold_21:  140672..138847 | scaffold_21:  141953..144466 | scaffold_21:  145140..147242 | scaffold_14:  871679..869242 | scaffold_2:  102427..100044 |
| *D. ananassae* | scaffold_12929:  2613731..2611533 | - | scaffold_12929:  2615638..2618534 | scaffold_13340:  22981806..22987992scaffold_13340:  22989446..22995839 | scaffold_12916:  10254679..10257058 |
| *D. mojavensis* | scaffold_6328:  1896082..1893301 | - | scaffold_6328:  1897850..1899964 | scaffold_6540:  13062148..13064577 | scaffold_6500:  24871475..24869082 |
| *D. virilis* | scaffold_12928:  917433..914559 | - | scaffold_12928:  918663..920639 | scaffold_13047:  18360884..18363309 | scaffold_12963:  5748510..5746126 |
| *D. pseudoobscura* | XL_group1a: 3128002..3129015 | - | XL_group1a: 3130032..3132128 | 2:  12921037..12926779 | Chromosome 4 |
| *D. persimilis* | scaffold_12:  149221..151091 | - | scaffold_12:  148234..146249 | scaffold_0:  7529608..7535326 | scaffold_5:  5468798..5466413 |
| *D. willistoni* | scaffold2_1100000004590:  4175231..4171619 | - | scaffold2_1100000004590:  4177328..4179574 | scaffold2_1100000004902:  6483677..6489830  scaffold2_1100000004902:  6488341..6494494 | scaffold2_1100000004884:  1033973..1036363 |
| *D. grimshawi* | scaffold_14853:  7615049..7617823 | - | scaffold_14853:  7614044..7612134 | scaffold_15074:  6337244..6343364  scaffold_15074:  6333402..6339555 | scaffold_15252:  7705266..7707660 |
