# Supplementary material for: The non-dosage compensated Lsp1α gene of Drosophila melanogaster escapes acetylation by MOF in larval fat body nuclei, but is flanked by two dosage compensated genes
Source: BMC Mol Biol. 2007 May 19;8:35. doi: 10.1186/1471-2199-8-35 (PMC1890558; doi:10.1186/1471-2199-8-35)
Supplement: Additional file 3 — Lsp1 gene identification in Drosophila species. [file 1471-2199-8-35-S3.doc]

**Additional file 3- *Lsp1* gene identification in *Drosophila*** species.

| *D. melanogaster* | *Lsp1α* | *Lsp1β* | *Lsp1γ* |
| --- | --- | --- | --- |
| *D. erecta* | Dere_GLEANR_HI_002240 | Dere_GLEANR_HI_007785 | Dere_GLEANR_HI_011867 |
| *D. yakuba* | Dyak_GLEANR_HI_013297 | Dyak_GLEANR_HI_001311 | Dyak_GLEANR_HI_003850 |
| *D. simulans* | - | - | - |
| *D. sechellia* | Dsec_GLEANR_HI_008345 | Dsec_GLEANR_HI_012111 | Dsec_GLEANR_HI_010287 |
| *D. ananassae* | Dana_GLEANR_HI_012191 | Dana_GLEANR_HI_009620 | Dana_GLEANR_HI_008146 |
| *D. mojavensis* | - | Dmoj_GLEANR_HI_006002 | Dmoj_GLEANR_HI_002081 |
| *D. virilis* | - | Dvir_GLEANR_HI_005415 | Dvir_GLEANR_HI_001310 |
| *D. pseudoobscura* | - | - | - |
| *D. persimilis* | - | Dper_GLEANR_HI_002729 | Dper_GLEANR_HI_004864 |
| *D. willistoni* | Dwil_GLEANR_HI_007113 | Dwil_GLEANR_HI_006388 | Dwil_GLEANR_HI_012053 |
| *D. grimshawi* | Dgri_GLEANR_HI_000692 | Dgri_GLEANR_HI_000691 | Dgri_GLEANR_HI_007461 |
