# Supplementary material for: The non-dosage compensated Lsp1α gene of Drosophila melanogaster escapes acetylation by MOF in larval fat body nuclei, but is flanked by two dosage compensated genes
Source: BMC Mol Biol. 2007 May 19;8:35. doi: 10.1186/1471-2199-8-35 (PMC1890558; doi:10.1186/1471-2199-8-35)

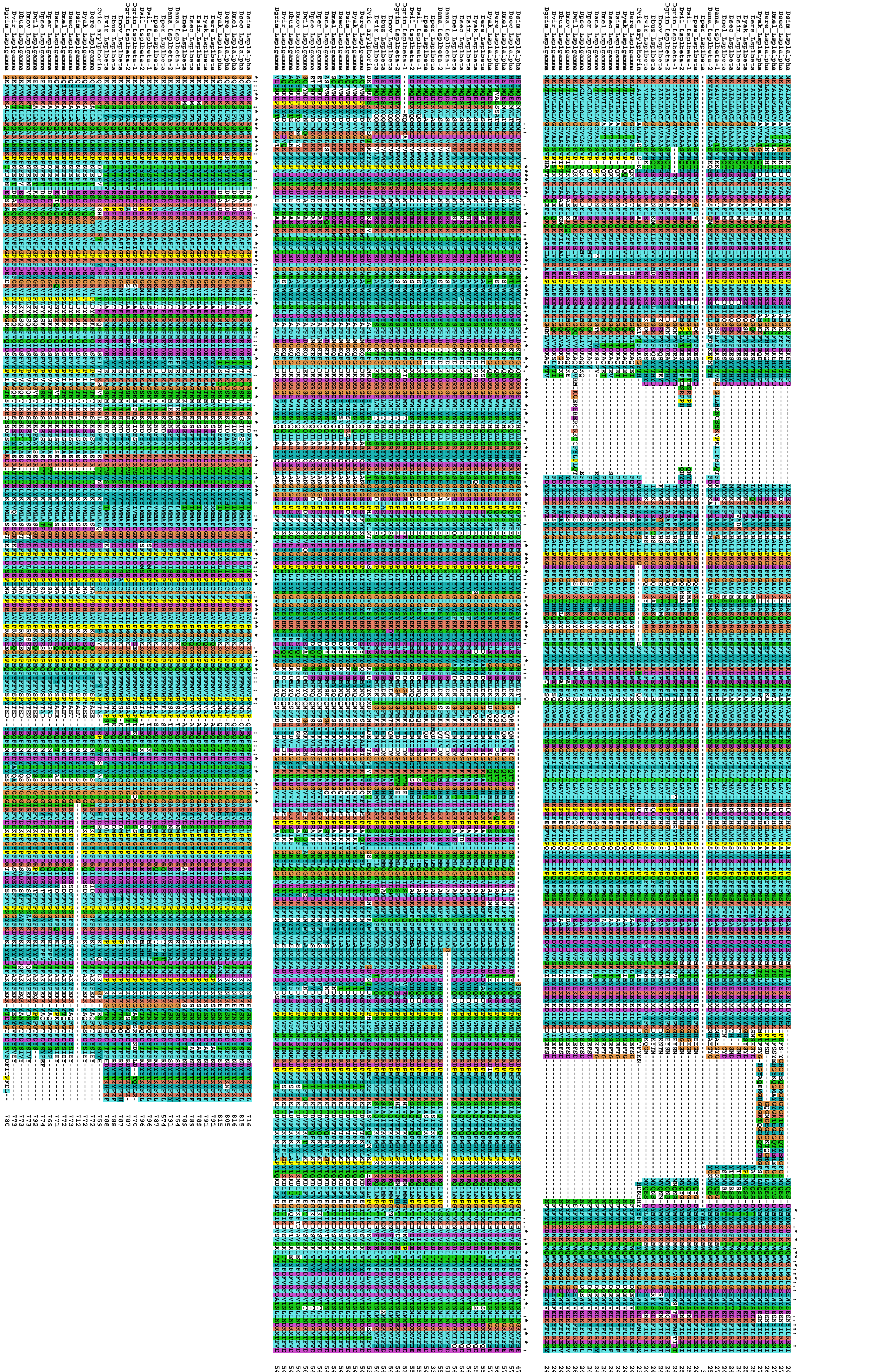

Supplement: Additional file 4 — Clustal X alignment of Drosophila Lsp1 sequences and Lsp1-like protein arylphorin from C. vicina. [file 1471-2199-8-35-S4.pdf]
